# Yield, Esterification Degree and Molecular Weight Evaluation of Pectins Isolated from Orange and Grapefruit Peels under Different Conditions

**DOI:** 10.1371/journal.pone.0161751

**Published:** 2016-09-19

**Authors:** Mohamed Yassine Sayah, Rachida Chabir, Hamid Benyahia, Youssef Rodi Kandri, Fouad Ouazzani Chahdi, Hanan Touzani, Faouzi Errachidi

**Affiliations:** 1 Laboratory of Applied Organic Chemistry, University Sidi Mohamed Ben Abdellah, Faculty of Science and Technology, Fes, Morocco; 2 Laboratory of Pathophysiology and Nutrition, Faculty of Medicine and Pharmacy, University Sidi Mohamed Ben Abdelah, Fes, Morocco; 3 Laboratoire d'Amélioration et Biotechnologie des Agrumes Institut National de La Recherche Agronomique (INRA) Kenitra, Maroc; 4 Laboratory of Physiology and Molecular Genetics, University Hassan II Ain Chock Faculty of Sciences, Casablanca, Morocco; The University of Tokyo, JAPAN

## Abstract

Orange (*Citrus sinensis*) and grapefruit (*Citrus paradise*) peels were used as a source of pectin, which was extracted under different conditions. The peels are used under two states: fresh and residual (after essential oil extraction). Organic acid (citric acid) and mineral acid (sulfuric acid) were used in the pectin extraction. The aim of this study is the evaluation the effect of extraction conditions on pectin yield, degree of esterification “DE” and on molecular weight “Mw”. Results showed that the pectin yield was higher using the residual peels. Moreover, both peels allow the obtainment of a high methoxyl pectin with DE >50%. The molecular weight was calculated using Mark-Houwink-Sakurada equation which describes its relationship with intrinsic viscosity. This later was determined using four equations; Huggins equation, kramer, Schulz-Blaschke and Martin equation. The molecular weight varied from 1.538 x10^05^ to 2.47x10^05^ g/mol for grapefruit pectin and from 1.639 x10^05^ to 2.471 x10^05^ g/mol for orange pectin.

## Introduction

Pectin substances are present in practically all fruits and vegetables. These substances are the major component of the middle lamella and of the primary cell walls of fruit tissues [1]. Many works reported that citrus pectin have inhibitory effects on fibroblast growth factor signal transduction [2,3], suppression of LPS-induced inflammatory responses [4] and preventive effect on cancer growth and metastasis [5–7]. Pectin has also several physiological and biological functions, such as stimulation of phagocytes and macrophages [8,9], spleen cells proliferation [10] and reduction of serum cholesterol [11]. Citrus peels are reported to be good source of pectin [12] which is widely used in the food industry for it gel-forming properties which depends on its degrees of methyl esterification DE and molecular weight [13]. The primary structural feature of this polysaccharides is a linear chain of poly-α-(1→4)-D-galacturonic acid with varying degrees of methyl esterification (DE). Commercial pectin preparations are divided into low-methoxyl (LM) and high-methoxyl (HM) pectins according to the degree of esterification (DE). Pectins with DE less than 50% are considered to be LM pectins [14,15]. Viscous-flow properties are very important during the production and applications of pectin and the higher the molecular weight is, the higher is its viscosity, the better is its grade [16,17]. Viscosity is affected by molecular weight, degree of methylation, concentration and temperature [17–19]. Usually, the extraction of the pectin is achieved by acid treatment at high temperature, using hydrochloric acid, nitric acid or sulfuric acid. This treatment allows the extraction and the solubilization of the pectin. However, some degradation reaction such as de-esterification and depolymerization will occur. Therefore, the extraction conditions (temperature, time, and pH) should be carefully controlled to achieve the desired pectin quality. Pectin is recovered by filtration or centrifugation process. Then, pectin is separated from the purified extract by precipitation using alcohol or by insoluble salt. The pectin is washed with alcohol to remove all impurities and finally dried and milled. Various alternative or complementary extraction processes have been suggested to improve the manufacture of pectin. We cite the extrusion pretreatment of the raw material when pectin is extracted from apple pomaces [20], Ultrasonic pulsation treatment in aqueous acidic solution which allow the reduction the processing time [21] and steam injection heating under pressure [22].

The objectives of this work is the determination of pectin yield, esterification degree and the molecular weight of orange and grapefruit peels pectin extracted after juice extraction, and from the residual peel after steam distillation using two kinds of acids: a mineral one which is the sulfuric acid, and organic one which is the citric acid.

## Results and Discussion

### 2.1 Pectin yield

**Fig 1:**
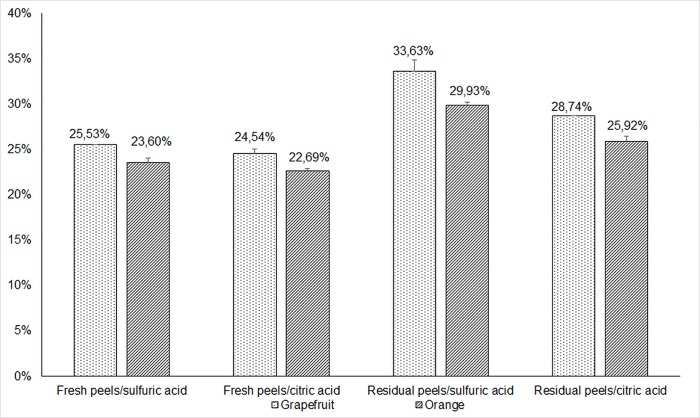
Effect of acids types and citrus peels stat on pectin yield.

According to the extraction process described in the Fig 1, the pectin yield obtained from the two citrus species (orange and grapefruit):

Based on a dry weight and all citrus peels states, grapefruit peels pectin yield was higher than that obtained from orange peels used as raw material. For both citrus species, the highest pectin yield was obtained using the residual peels. Residual Orange peels pectin yield was 29.93% and 25.92% using sulfuric and citric acid respectively, while the fresh peels give the lowest pectin yield; 23.60% using sulfuric acid and 22.69% using citric acid. The highest pectin yield obtained from grapefruit peels was 33.63% from residual one using sulfuric acid, while using citric acid gives 28.74% as pectin yield. The pectin yield obtained from fresh grapefruit peels was 25.53% and 24.54% when using sulfuric and citric acid respectively. The increase in pectin yield in both orange and grapefruit peels is ranging from 3.23% to 8.10%. The increase in pectin yield is noticed when residual peels were used can be explained by the thermal treatment during the hydro-distillation which weakened the structure of the peels thus increasing interaction between acidic solution and raw material during the extraction, therefore leading to an effective increase of pectin yield. In the process of orange essential oil and pectin extraction, it has been recommended to first extract oil using simple distillation and then isolate pectin with acid hydrolysis technique which may lead to 46.46% as pectin yield [23]. Kar has removed essential oil form orange peels using petroleum ether and used these peels as raw material for pectin extraction. The yield obtained using hydrochloric acid was 29.58% [17]. Bagherian found that the pectin yield was 19.16% using a conventional method extraction on grapefruit peels [24].

### 2.2 Degree of esterification

The DE of grapefruit peels pectin ranged from 70.73±1.33% to 75.53±0.95% (Table 1) and ranged from 63.29±0.84% to 75.00±0.53% for orange peels pectin. Based on DE, pectin can be classified as high methoxyl pectin with DE >50% which is commercially available food-grade high methoxyl pectin [24,25].

**Table 1:** Degree of esterification of orange and grapefruit pectins.

Citrus peels	Peels stats	Acids	DE%
Grapefruit	Fresh	Sulfuric	71.72±1.06%
		Citric	70.73±1.33%
	Residual	Sulfuric	74.49±1.2%
		Citric	75.53±0.95%
Orange	Fresh	Sulfuric	63.29±0.84%
		Citric	65.49±0.57%
	Residual	Sulfuric	74.51±0.41%
		Citric	75.00±0.53%

From Table 1 we notice that the degree of esterification increases when we use the residual peels instead of fresh ones for pectin extraction and the DE was higher when the pectin is extracted using citric acid. The temperature and the acid concentration contribute to increase the DE of pectin [24,26]. The thermal treatment of the peels undergone during essential oils extraction affects the pectin degrees of esterification. Indeed harsh temperature conditions increases the degree of esterification [27,28]. Generally and for most of the pectins, it appears that the citric acid has a positive effect on the degree of esterification compared with that of sulfuric acid. This positive effect of citric acid has been noticed in various works [29,30].

### 2.3 Intrinsic viscosity determination

**Fig 2:**
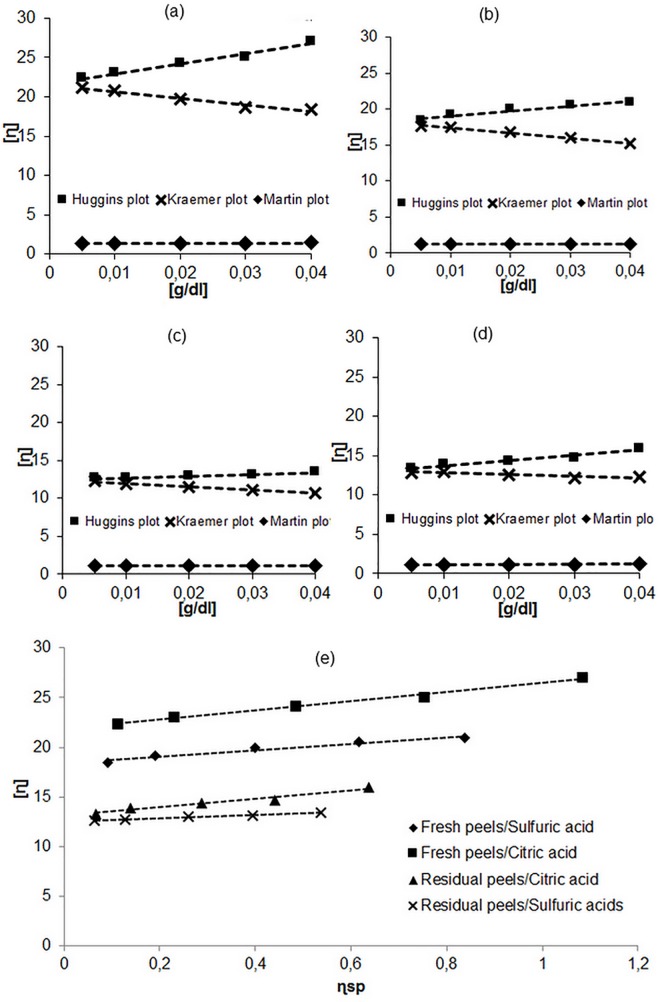
Huggins, Kraemer and Martin plots for grapefruit pectin extracted from fresh peels using sulfuric acid (a) and citric acid (b), and from residual peels using sulfuric acid (c) and citric acid (d)and Schulz-Blacschke plot of all grapefruit pectin solutions (e).

**Fig 3:**
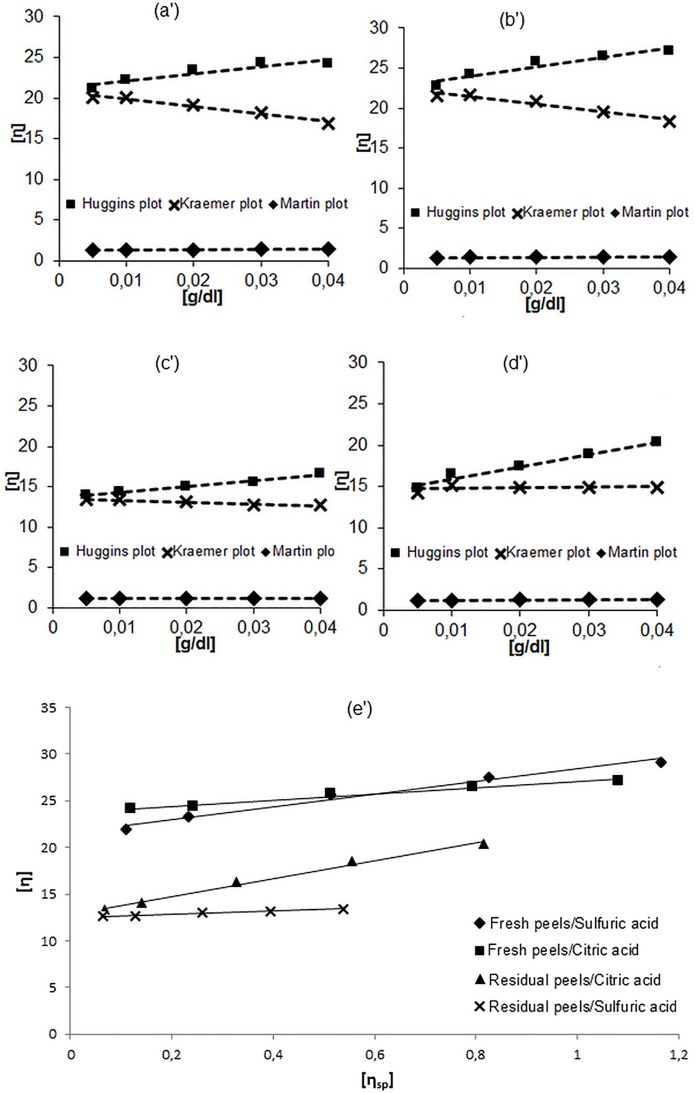
Huggins, Kraemer, and Martin plots for orange pectin extracted from fresh peels using sulfuric acid (a’), and citric acid (b’) and from residual peels using sulfuric acid (c’), and citric acid (d’) and Schulz-Blacschke plot of all orange pectin solutions (e’)

Intrinsic viscosity of a polymer solution is the assessment of polymer capacity to enhance viscosity [17]. Figs 2 and 3 show the method adopted for intrinsic viscosity determination. The later can be obtained using a linear regression graphic double-extrapolation procedure which involves extrapolating the course of a specific viscosity to infinite dilution [31]. As shown in Figs 2 and 3, the Huggins and Kraemer plots have a high level of linearity and extrapolate approximately to the same intercept at zero concentration for all extracted citrus pectins. These results suggest that the interference of ionic strength effects and molecular aggregation on viscosity behavior were reduced by the good choice of sodium chloride as solvent. In order to confirm and compare the results obtained from Huggins and Kraemer plots, values of intrinsic viscosity were also compared with those obtained by plotting Schulz-Blaschke Eq (8), and Martin Eq (9). The values of the intrinsic viscosity were comparable to each other and are also comparable to those obtained from Huggins and Kraemer plots for each pectin solution. For each pectin solution, the intrinsic viscosity was determined using the linear regression graphic extrapolation of the four equation mentioned Materials and Methods section (6, 7, 8 and 9). The values of intrinsic viscosity of all pectin solutions deduced from the plots are presented in Table 2 for grapefruit and Table 3 for orange peels.

**Table 2:** Intrinsic viscosity of grapefruit pectin solutions.

	Intrinsic viscosity [η] (dl/g)				
Grapefruit peels Stats/Acid	Huggins plot	Kraemers plot	Schulz-Blaschke plot	Martin plot	average value of [η] (dl/g)
Fresh peels/Sulfuric acid	18.385	18.173	18.454	18.412	18.356±0.091^a^
Fresh peels/Citric acid	21.231	21.332	21.691	21.449	21.426±0.144^b^
Residual peels/Sulfuric acid	12.512	12.426	12.524	12.517	12.495±0.034^c^
Residual peels/Citric acid	13.015	13.051	13.106	13.071	13.061±0.028^d^

**Table 3:** Intrinsic viscosity of orange pectin solutions.

	Intrinsic viscosity [η] (dl/g)				
Orange peels Stats/Acid	Huggins plot	Kraemers plot	Schulz-Blaschke plot	Martin plot	Average value of [η] (dl/g)
**Fresh peels/Sulfuric acid**	20.687	20.961	21.361	20.980	20.997±0.18^**a**^
**Fresh peels/Citric acid**	22,874	22,479	22,906	22,699	22,739±0,15^**b**^
**Residual peels/ Sulfuric acid**	13.556	13.595	13.665	13.618	13.608±0.03^**c**^
**Residual peels/ Citric acid**	14.414	14.638	14.771	14.581	14.601±0.1^**d**^

The ANOVA test showed that the difference between all the peels stats/Acid is significant, F ratio was 5059.83 at (p>0.05). In order to compare each pair of peels stats/Acid we performed Tukey-Kramer HSD (honestly significant difference) test. Results showed that peels stats/Acid that are not connected by the same letter (a, b, c and d) are significantly different.

High degree of linearity was observed for all the plots. It shows that Huggins’s equation, Kraemer’s equation, the Schulz-Blaschke and Martin’s equation are suitable to be applied to calculate the intrinsic viscosity [η] for all grapefruit pectin solutions (Fig 2) and also for orange pectin solutions (Fig 3). Moreover, the extrapolation plots of the four mentioned equations give approximately the same value of the intrinsic viscosity. From Table 2 and the Table 3, the intrinsic viscosity values depend on the nature of the acid used in the extraction of pectin and on the peels’ states. It was seen that citric acid gives a high intrinsic viscosity value than that obtained using sulfuric acid, except for orange residual peels, where sulfuric acid extracted pectin with higher intrinsic viscosity than that obtained using citric acid. In addition, both of grapefruit and orange fresh peels give pectin with higher intrinsic viscosity than residual peels.

The ANOVA test showed that the difference between the different pectin molecular weight is significant, the F ratio was 2387.67, at P<0.05. The Tukey-Kramer HSD test showed that all peels stats/ acid were significantly different and that none was connected by the same latter.

**Fig 4:**
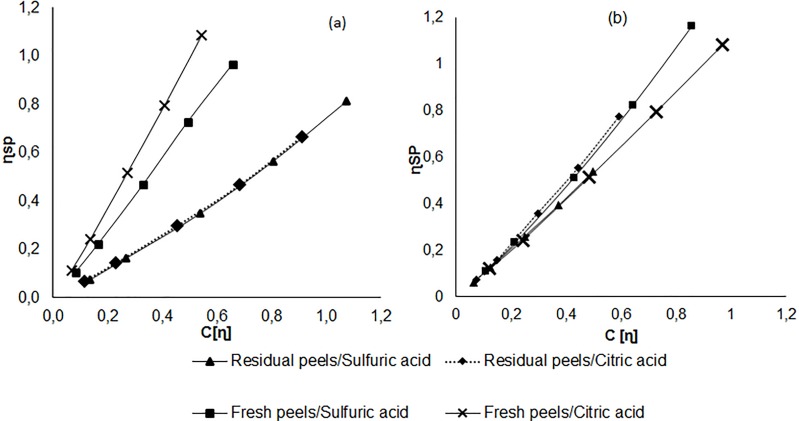
Variation of “zero shear” specific viscosity (ɳ_sp_) with degree of space-occupancy (c[ɳ]) for Orange (a) and Grapefruit (b) pectins.

To get more validity to our results, and according to Evageliou [32], we analyzed pectin chains behavior while flowing through the viscometer, by studying the variation of “zero shear” specific viscosity (η_sp_) with degree of space-occupancy (C[η]). The concentration at which the total volume occupied by the polymer becomes equal to the total volume of the solution is known as the overlap concentration C*. At this concentration polymer entanglement my happen [33]. Theoretical calculations showed that C* is reached when c[η] = 1. Thus the polymer solution is defined to be dilute when C < C* and at higher concentrations (C > C*) the polymer is termed semi-dilute. When C > C* an entangled network can be formed and a chain movement occur by a difficult process that changes the solution properties [32]. However for many polysaccharides significant restriction to the movement of individual chain occur at C = 4C* [32,34]. Fig 4 shows that all pectin solutions are termed as dilute solutions for both citrus species used in this work (a: orange; b: grapefruit) and that none of the solutions reached the four time coil-overlap concentration that causes significant changes in solution properties.

All pectin solutions are defined as dilute solutions (C < C*). The specific viscosity is proportional to the parameter C [ɳ] and it is scaled linearly. In addition, the range of concentrations used in intrinsic viscosity determination does not exceed the critical concentration C* beyond which the overlap between pectin chains are likely to occur, thereby distorting the measurement of the viscosity of pectin solutions. Various polysaccharides were studied to determine the concentration at which the behavior of their solution becomes messy because of overlaps polysaccharide chains. As a result, the transition from dilute solution behavior to the behavior of concentrated solution occurs when the specific viscosity ɳ_sp_ ≈ 10 and the parameter C [ɳ] = 4 C* [35]. All pectin solutions are dilute solutions because they have a specific viscosity of not more than 1.5. Also the parameter “C [ɳ]”, which informs about the occupation state of volume of the solvent by the polymer, is well below the threshold 4C*. Beyond the threshold of 4 C*, the pectin solution is defined to be concentrated. Therefore, there are overlaps that cause significant restraints on individual pectin chains’ movement relative to each other during the flow of the pectin solution through the capillary viscometer. Thus distorting the viscosity measurements.

### 2.4 Molecular weight determination

Since Huggins’s equation, Kraemer’s equation, the Schulz-Blaschke and Martin equation can be used to determinate the intrinsic viscosity we calculated the average of all intrinsic viscosity gotten by the Eqs (6, 7, 8 & 9) and we used this average intrinsic viscosity value in the Mark-Houwink-Sakurada Eq (10) to calculate the molecular weight Mw (Table 4). The molecular weight of pectin extracted from the residual citrus peels, after essential oil distillation, was lower than the one obtained using fresh peels for both citrus species. It can be explained by the thermal degradation of the pectin during the essential oil extraction, which has a lowering effect on pectin molecular weight. Bagherian and Fishman reported that continued heating of pectin may lead to pectin networks disaggregation, thus decreasing the molecular weight [24,36].

**Table 4:** Orange and grapefruit pectin molecular weight (Mw).

	Mw (g/mol)	
Peels stats/acid used	Grapfruit	Orange
Fresh peels/ sulfuric	2.300 x10^05^	2.266 x10^05^
Fresh peels/ citric	2.472 x10^05^	2.405 x10^05^
Residual peels/sulfuric	1.538 x10^05^	1.639 x10^05^
Residual peels/citric	1.544 x10^05^	1.728 x10^05^

To support our results, we performed an exclusion chromatography using Sephadex G-150 as stationary phase. We hydrate the Sephadex (5g) with water (100ml) for one hour. After filling the column (15cm x 1.4cm), we determined the retention time of the following polymers:

- Sodium carboxymethyl cellulose (SIGMA-ALDRICH, Mw ~90000 g/mol)
- Commercial Orange pectin (SIGMA-ALDRICH, Mw = 195000—DE = 70%)
- Locust bean gum (SIGMA-ALDRICH, MW 315000 g/mol)

A total of 17 fractions (each 15 seconds) were collected and for the revelation of the polymers presence we used the Dubois protocol [37]. This protocol allows the colorimetric determination of sugar and related substances. The aim of this experiment is obtaining a calibration curve that links the molecular weight to the retention time. The link between the molecular weight and the retention time is represented as the following equation of the calibration curve Rt = a Mw+b (Rt: retention time; Mw: Molecular weight). The equation of the calibration curve is:

$$y=-0.0009x+292.19\;\text{and}\;r^{2}=0.9711.$$

The presence of the polymers was detected using Dubois protocol. While the intensity of the peak was evaluated using the absorbance reading of each fraction. After the establishment of the calibration curve, we performed the exclusion chromatography of our orange pectin which was extracted from fresh peel using citric acid (Mw = 2.405*10^05^ g/mol) and from residual peels using sulfuric acid (Mw = 1.639*10^05^ g/mol respectively) in order to confirm their molecular weights. Table 5 shows the results obtained according to the sampling interval:

**Table 5:** Retention time Orange pectin.

	Orange pectin	
Retention time (s)	Fresh peels / Citric acid	Residual peels / Sulfuric acid
0	0	0
15	0	0
30	0,034	0
45	0,067	0
60	0,122	00
75	0,156	0,02
90	0,286	0,04
105	0,201	0,07
120	0,166	0,11
135	0,124	0,16
150	0,091	0,23
165	0,042	0,16
180	0,012	0,12
195	0	0,08
210	0	0,06
225	0	0,03

Based on the absorbance, the peak for pectin which was extracted from fresh peels using citric acid is at 90 seconds, and at 150 seconds for the pectin which was extracted from residual peels using sulfuric acid. Using the calibration curve equation we found that the molecular weight of the orange pectin (fresh peels/citric acid) is 1.580*10^5^ g/mol and 2.247*10^5^ g/mol for the second orange pectin (residual peels / sulfuric acid). According to the viscometric measurements, the molecular weight of the orange pectin which was extracted from fresh peels using citric acid is 2.405*10^5^ g/mol while it was 1.639*10^05^ g/mol for the pectin which was extracted from residual peels using sulfuric acid. These results are close to the ones obtained by viscometric measurements, thus supporting them.

The use of the sulfuric acid gives pectin with a lower molecular weight than citric acid in grapefruit peels case. It was found that pectin extracted from orange had nearly the same quality as the one obtained from grapefruit based on Mw. According to Zhou, residual orange peels (after the extraction of essential oil and flavonoids) provides pectin with molecular weight of 1.65*10^5^ g/mol [38]. This result is less than what we’ve got using our residual orange peels 1.728 *10^5^ g/mol using citric acid and nearly the same pectin molecular weight in the case of the sulfuric acid and 1.639*10^5^ g/mol. The molecular weight can vary depending on the extraction protocol conditions and the state of raw material. Haring found that the molecular weight of citrus pectin varied from 2 x10^4^ to 2 x10^5^ g/mol [39]. Morris found that the molecular weight of commercial pectin, with different esterification degree, was approximately constant in the range of 1.9 x10^5^±3 x10^4^ g/mol [40] and 1.95 x10^5^±5 x10^3^ g/mol [41]. Our results show that we were able to extract pectin that equals the commercial one based on molecular weight and even goes beyond it as in the case of pectin extracted from the fresh peels. This molecular weight decreases for pectin extracted from residual peels. High temperature and long extraction leads to a drop in the molecular weight of the pectin obtained [24,28]. In our case, this negative effect of temperature begins during the extraction of essential oils. This temperature has a positive effect on pectin yield certainly, but its impact on pectin molecular weight is negative.

## Conclusion

Pectin quality varies according to the citrus species waste used as raw material. The nature of the acid used in extraction affects significantly pectin yield and the molecular weight. Residual citrus peels provide a high yield of pectin due to the thermal treatment undergone during the essential oil distillation, which weakens the primary cell walls, thus improving pectin recovery. In addition, the degree of esterification observed for all extracted pectin was higher than 50%, which classifies them as high methoxyl pectin. Pectin extracted from fresh peels had a better grade than the one extracted from residual peels in terms of molecular weight, but it does not prohibit its food industry utilization. Having pectin with a high molecular weight is important because it is possible to carry out controlled de-polymerizations reactions that allow the obtainment of low molecular weight pectin. Pectin with a low molecular weight has several benefits for the human body. Pectin consumption can potentially play an important role in detoxification of harmful chemicals, toxins and heavy metals in the body. This property makes the pectin an attractive option to treat heavy metal intoxication. Because of its large molecular weight, pectin cannot pass into the blood system. This passage is made possible by reducing the molecular weight pectin allowing it to express its chelating and detoxifying power in the human body. [42,43].

## Materials and Methods

### 4.1 Raw Materials

In the present work, two citrus species were used: grapefruit (*Citrus paradisi*) and orange (*Citrus sinensis*). These citrus peels were collected after juice extraction process and were treated according to the method described below.

### 4.2 Simple preparation

In previous works [44], we have used two batches of ground citrus peels. The first batch was washed with water in order to remove impurities, dried and ground for pectin extraction, while essential oil were extracted from the second one (vapodistillation). Pectin extraction

In a previous work [45] we have optimized the pectin extraction conditions by targeting: time, temperature and acid concentration to get the highest pectin yield. We used these optimal conditions (0.1M; 80°C; 60min) in pectin extraction for its characterization. Pectin was extracted from both citrus peels states mentioned above, with aqueous sulfuric acid and citric acid (1:30, w/v) under reflux. After centrifugation (3000g for 10 min), each acid extract was filtered and the pectin was precipitated with two volumes of ethanol 96%, stirred slowly and stored in a refrigerator overnight in order to fully achieve pectin precipitation [46,47]. Afterward, the gelatinous precipitate was removed by centrifugation, washed three times with 96% ethanol to remove the monosaccharides and disaccharides [48]. The wet pectin was dried under vacuum and the weight was monitored until stabilization. All the experiments were done in triplicate and results were reproducible with an acceptable average error.

### 4.3 Pectin yield

Pectin yield was calculated as follows:

$$Pectin\;yield\;\left(\%\right)=\left(m_{0}/m\right)*100$$ (1)

“m_0_” (g) is the dried pectin weight and “m” (g) is the dried raw material weight.

### 4.4 Degree of esterification (DE)

The DE of the pectin was determined by the titrimetric method [49] with minor modifications. The dehydrated sample was moistened with 2 mL of ethanol and dissolved in 25 mL of distilled water (free of carbon dioxide). Two drops of phenolphthalein were added after complete dissolution of the sample, then we started the titration process with 0.25 M sodium hydroxide to neutralize the free carboxyl acids from anhydro-galacturonic acid and the result was recorded as (V1). Afterward, 10 mL of 0.25 M sodium hydroxide was added and stirred for 30 min for hydrolysis, followed by the addition of 10 mL of 0.25 M hydrochloric acid and stirring until the complete disappearance of the pink color of the solution. HCl Excess was titrated with 0.1 N NaOH. The number of the esterified carboxyl groups was calculated from the volume of 0.1 NaOH solution spent for titration (V2). The DE of the pectin was calculated using the following formula:

$$\%DE=\frac{V_{2}}{V_{2}+V_{1}}*100$$ (2)

### 4.5 Viscosity measurement

For polyelectrolytes such as pectin, there is a progressive reduction in coil volume with increasing ionic strength [50]. When solutions are prepared in water, the ionic strength changes as the polymer concentration does, with a consequent variation in coil dimensions. Meaningful values of intrinsic viscosity can be obtained only if the ionic strength is maintained constant by adding extraneous salt. pectin solution (0.05, 0.1, 0.2, 0.3, and 0.4 kg/m^3^) was prepared by dissolving it in 0.1 mol/L sodium chloride solution to reduce the electro-viscous effect to a minimum [38]. The mixture was then heated to 25°C and allowed to stand with mixing at ambient temperature for 12 h. After filtration, 15 ml of pectin solutions were pipetted into the capillary (Cannon-fenske) viscometer for viscosity measurements and was immersed in a thermostatic water bath at 25.0°C. The pectin solution was loaded into the viscometer and allowed to equilibrate at the bath temperature (25°C) before starting the experiment. The time for the sample to flow from one level indicator to another, known as flow time, was measured and converted to kinematic viscosity and the densities of solutions were measured using a pycnometer. All of the experiments were triplicated and the average values were taken with an acceptable average error.

### 4.6 Intrinsic viscosity measurements

To determinate the intrinsic viscosity, the following steps and notions are very significant:

The relative viscosity was calculated using the following equation [4,51,52] and because of the low concentration used d/d_s_ was taken as unity:

$$\eta_{r}=\frac{\eta }{\eta_{s}}=\frac{t\;d}{t_{s}d_{s}}$$ (3)

Where “η_r_” the relative viscosity, η the viscosity of pectin solution (Pas), “η_s_” the viscosity of solvent (Pas), “t” the time taken by solvent to flow in viscometer (s). “t_s_” the time taken by solution to flow in viscometer (s). “ds” the density of solution (kg/m^3^). “d_s_” the density of solvent (0.1 mol/L sodium chloride).

Relative viscosity values were converted to specific viscosities (η_sp_) using the following equation [51]:

$$\eta_{sp}=\frac{\eta -\eta_{s}}{\eta_{s}}=\eta_{r}-1$$ (4)

In dilute solutions, that is, in conditions of negligible interactions between pectin chains, the intrinsic viscosity [ɳ] of the biopolymer depends only on the dimensions of the polymer chain. The intrinsic viscosity [η] is defined as the limit of η_sp_/c or ln(η_r_/c) as the concentration approaches zero and the principal determination method of the intrinsic viscosity magnitude is to extrapolate the reduced viscosity to its value at zero solute concentration [53–55].

$$\left[ɳ\right]=\lim_{c\to 0}\frac{\eta_{sp}}{\text{c}}=\lim_{c\to 0}\frac{ln\;\eta_{r}}{\text{c}}$$ (5)

Where “c” is the concentration of pectin solution and η_sp_ is the specific viscosity.

If the plot of reduced viscosity η_red_ versus concentration shows a linear trend, the Huggins Eq (6) can be used to calculate intrinsic viscosity from the intercept, and for the kramer plot using its Eq (7), where a handled value of inherent viscosity η_inh_ is plot versus solution concentration [54,56,57]:

$$\text{Huggins equation}\;:\;\eta_{red}=\frac{\eta_{sp}}{\text{c}}={\left[ɳ\right]}^{2}K_{H\;}C+\left[ɳ\right]$$ (6)

$$\text{Kramer equation}:\;\eta_{inh}=\frac{\ln\left(\eta_{r}\right)}{\text{c}}={\left[ɳ\right]}^{2}K_{K\;}C+\left[ɳ\right]$$ (7)

Moreover, the following equations were used to obtain [η] [58]:

$$\text{Schulz-Blaschke equation}:\;\frac{\eta_{sp}}{\text{c}}=\left[ɳ\right]K_{SB\;}\eta_{sp}+\left[ɳ\right]$$ (8)

$$\text{Martin equation}:\;Log\left(\frac{\eta_{sp}}{\text{c}}\right)=\left[ɳ\right]cK^{"}+Log\left[ɳ\right]$$ (9)

### 4.7 Determination of molecular weight

Intrinsic viscosity is one of the most important characteristic of polymers. It depends only on the molecular mass when the sample’s measuring conditions (solvent and temperature) are set. The Mark-Houwink-Sakurada equation describes the relationship between intrinsic viscosity and molecular weight *Mw*.

$$\left[ɳ\right]=\text{K}M_{w}^{\alpha }$$ (10)

Both K and α depend on temperature, solute and solvent characteristics. A large number of models have been used to deduce [η]-*Mw* relationships. In this work the following values were assumed K = 1.4*10^−6^ and α = 1.43 [38].

### 4.8 Statistical analysis

For a statistical analysis, the variance analysis (ANOVA) was used to treat different averages obtained. Data was analyzed by the analysis of variance, and averages were separated by the least significant difference when significant F (P<5%) values were observed.

## Supporting Information

S1 Fig Fig A of S1 Fig. Effect of acids types and citrus peels stat on pectin yield. Fig B of S1 Fig. Huggins, Kraemer and Martin plots for grapefruit pectin extracted from fresh peels using sulfuric acid (a) and citric acid (b), and from residual peels using sulfuric acid (c) and citric acid (d) and Schulz-Blacschke plot of all grapefruit pectin solutions (e). Fig C of S1 Fig. Huggins, Kraemer, and Martin plots for orange pectin extracted from fresh peels using sulfuric acid (a’), and citric acid (b’) and from residual peels using sulfuric acid (c’), and citric acid (d’) and Schulz-Blacschke plot of all orange pectin solutions (e’). Fig D of S1 Fig. Variation of “zero shear” specific viscosity (ɳ_sp_) with degree of space-occupancy (c[ɳ]) for Orange (a) and Grapefruit (b) pectins.
(XLSX)

S1 Table Table A of S1 Table. Degree of esterification of orange and grapefruit pectins. Table B of S1 Table. Intrinsic viscosity of grapefruit pectin solutions. Table C of S1 Table. Intrinsic viscosity of orange pectin solutions. Table D of S1 Table. Orange and grapefruit pectin molecular weight (Mw). Table E of S1 Table. Retention time Orange pectin.
(XLSX)
